# Signaling Crosstalk of TGF-β/ALK5 and PAR2/PAR1: A Complex Regulatory Network Controlling Fibrosis and Cancer

**DOI:** 10.3390/ijms19061568

**Published:** 2018-05-24

**Authors:** Hendrik Ungefroren, Frank Gieseler, Roland Kaufmann, Utz Settmacher, Hendrik Lehnert, Bernhard H. Rauch

**Affiliations:** 1 First Department of Medicine, University Hospital Schleswig-Holstein, D-23538 Lübeck, Germany; frank.gieseler@uksh.de (F.G.); Hendrik.Lehnert@uni-luebeck.de (H.L.); 2 Department of General and Thoracic Surgery, University Hospital Schleswig-Holstein, D-24105 Kiel, Germany; 3 Department of General, Visceral and Vascular Surgery, Jena University Hospital, D-07747 Jena, Germany; Roland.Kaufmann@med.uni-jena.de (R.K.); Utz.Settmacher@med.uni-jena.de (U.S.); 4 Department of General Pharmacology, Institute of Pharmacology, University Medicine Greifswald, D-17487 Greifswald, Germany; Bernhard.Rauch@uni-greifswald.de

**Keywords:** TGF-β, PAR1, PAR2, ALK5, serine proteinases, signaling, fibrosis, cancer

## Abstract

Both signaling by transforming growth factor-β (TGF-β) and agonists of the G Protein-coupled receptors proteinase-activated receptor-1 (PAR1) and -2 (PAR2) have been linked to tissue fibrosis and cancer. Intriguingly, TGF-β and PAR signaling either converge on the regulation of certain matrix genes overexpressed in these pathologies or display mutual regulation of their signaling components, which is mediated in part through sphingosine kinases and sphingosine-1-phosphate and indicative of an intimate signaling crosstalk between the two pathways. In the first part of this review, we summarize the various regulatory interactions that have been discovered so far according to the organ/tissue in which they were described. In the second part, we highlight the types of signaling crosstalk between TGF-β on the one hand and PAR2/PAR1 on the other hand. Both ligand–receptor systems interact at various levels and by several mechanisms including mutual regulation of ligand–ligand, ligand–receptor, and receptor–receptor at the transcriptional, post-transcriptional, and receptor transactivation levels. These mutual interactions between PAR2/PAR1 and TGF-β signaling components eventually result in feed-forward loops/vicious cycles of matrix deposition and malignant traits that exacerbate fibrosis and oncogenesis, respectively. Given the crucial role of PAR2 and PAR1 in controlling TGF-β receptor activation, signaling, TGF-β synthesis and bioactivation, combining PAR inhibitors with TGF-β blocking agents may turn out to be more efficient than targeting TGF-β alone in alleviating unwanted TGF-β-dependent responses but retaining the beneficial ones.

## 1. Introduction

Transforming growth factor-β (TGF-β) and the G protein-coupled receptors “proteinase-activated receptor 1” (PAR1) and PAR2 represent crucial factors in tissue fibrosis and cancer development. Recent results from our and other laboratories have shown a previously unexpected array of functional interactions between TGF-β and PAR2 signaling and, to a lesser extent, PAR1 signaling, which eventually synergistically enhance fibrogenesis and tumorigenesis. The finding that both PARs crosstalk with TGF-β signaling may be because PAR2 and PAR1 physically interact with each other and share several signaling pathways and functional activities. Following a brief introduction of the major players, we summarize in the first part of this review the diverse regulatory interactions that have been discovered in (patho)physiological conditions such as fibrosis, wound healing, and cancer, and group them according to the affected tissue/organ. In the second part, we highlight the various mechanisms of signaling crosstalk between TGF-β on the one hand and PAR2 or PAR1 on the other hand.

## 2. TGF-β, PARs, and Sphingosine-1-Phosphate as a Mediator between TGF-β and PAR Signaling

### 2.1. TGF-β

The TGF-β superfamily consists of 33 members, most of which are dimeric, secreted polypeptides. In addition to the three TGF-β isoforms (The term “TGF-β” refers to all three TGF-β isoforms unless the specific isoform is indicated by the respective number, i.e. TGF-β1), TGF-β1, TGF-β2 and TGF-β3, this superfamily includes the activins, inhibins, bone morphogenetic proteins (BMPs), growth and differentiation factors (GDFs), myostatin, nodal, lefty, and mullerian inhibiting substance (MIS) [1]. The three TGF-β isoforms are structurally similar cytokines encoded by separate genes that act in an autocrine or paracrine manner to regulate early embryonic development, homeostasis and regeneration of adult tissues, as well as various pathologies [1]. They are secreted as inactive precursors bound to latency-associated peptides and need to be activated extracellularly by proteinases such as elastase and matrix metalloproteinases (MMPs), or through conformational changes induced by various integrins [2,3]. All three isoforms evoke their cellular effects on target cells by binding to transmembrane dual specificity receptors which possess strong serine/threonine kinase activity and weak tyrosine kinase activity [4]. The most important TGF-β receptor types are type I receptor (TβRI, also known as ALK5) and type II receptor (TβRII) both of which belong to the class of serine/threonine protein kinases. Ligand binding assembles a complex consisting of TβRII (signal-activating receptor) and ALK5 (signal-propagating receptor). In the complex, TβRII phosphorylates ALK5, which then propagates the signal via phosphorylation of substrate SMAD proteins, SMAD2 and SMAD3. The phosphorylated forms of these proteins bind to another SMAD, SMAD4, and the resulting complex translocates to the nucleus where it binds to regulatory regions of TGF-β target genes to drive their transcriptional activation [5,6,7,8,9,10]. However, this simple mode of signaling cannot explain how TGF-β can evoke such a diverse and sometimes opposite set of functional responses in a cell such as inhibition of cell proliferation or invasion in one cell type but promotion of cell growth or metastatic dissemination in another. These responses appear to be context-dependent and are shaped by three types of contextual determinants which underlie the pleiotropic capacity of TGF-β signaling. (i) The response of a cell is determined by the extracellular and intracellular anatomy of the TGF-β signal transduction system. The abundance and activity of different TGF-β ligands, receptors, and regulators determine the nature and intensity of the TGF-β signal in the nucleus. For instance, the levels of TGF-β receptors correlate with TGF-β responsiveness [11,12], hence regulators, such as microRNAs that control their expression determine initiation, duration, and intensity of signaling in response to TGF-β [13]. (ii) A second set of determinants comprises factors that cooperate with SMAD proteins to regulate transcription. Lineage-specific transcription factors direct TGF-β-activated SMAD proteins to specific loci in the various types of cells and the resulting SMAD complexes then recruit chromatin modifiers to regulate transcription. The availability of these SMAD partners determines what genes will be targeted and whether the outcome is activation or repression. (iii) Finally, the epigenetic landscape of the cell, characterized by DNA methylation patterns, histone modifications, nucleosome positioning, and non-coding RNAs, dictates what genes are sensitive to regulation. Besides these determinants, signal diversity may also result from modulatory interactions/crosstalk with other signaling pathways which are exerted at different levels of the signaling cascade. For instance, SMADs are phosphorylated in the linker region by kinases activated in response to tyrosine kinase receptors like the EGF receptor (EGFR) [14,15,16]. This non-canonical Smad signaling may block a particular SMAD-dependent response such as growth inhibition thereby enhancing the growth-promoting function of EGFR. Moreover, non-Smad signaling pathways involve the ALK5-dependent activation of mitogen-activated protein kinases (MAPKs) such as p38, jun-*N*-terminal kinase 1/2 (JNK1/2), or extracellular signal-regulated kinase 1/2 (ERK1/2). While the activation of ERK has been shown to occur through the tyrosine kinase function of ALK5 [4], p38 and JNK are phosphorylated on serine or threonine by the serine/threonine function of ALK5. The activation/phosphorylation of p38 by ALK5 can be either direct, which is associated with a rapid kinetics, or can occur downstream of Smad3 and intermediate induction of GADD45β resulting in a delayed type of activation [17,18]. P38 signaling has been shown to be involved in the TGF-β response of various matrix-associated and profibrotic genes [19,20].

TGF-β has strong profibrotic activity and promotes cancer progression. Since both fibrosis and certain types of cancer can develop from inflammatory events, coupling of TGF-β signaling to receptors with proinflammatory and tumor-promoting activity would be expected to enhance its profibrotic and protumorigenic functions. Of particular interest here are receptors with proinflammatory and proinvasive/metastatic potential such as the proteinase-activated receptors (PARs), PAR2 and PAR1.

### 2.2. PARs

PARs are a subfamily of G protein-coupled receptors (GPCRs) comprised of four members, PAR1–4, which play crucial roles in tissue hemostasis, thrombosis, wound healing, inflammation-associated disorders, fibrosis, and cancer. The activation of PARs involves receptor cleavage by different serine proteinases at specific sites within the extracellular N-terminal domain and the exposure of an N-terminal tethered ligand that binds to and activates the cleaved receptor. The prototype activator for PAR1 is thrombin and for PAR2 it is trypsin and FXa complexed with tissue factor (TF). The cleaved receptors then signal intracellularly via classical G proteins and β-arrestin [21,22,23]. In addition, different receptor crosstalk mechanisms contribute to a high diversity of PAR signal transduction that results in multiple physiological effects. PARs can homo- and heterodimerize, transactivate receptor tyrosine kinases, and communicate with other GPCRs, toll-like receptors, NOD-like receptors, ion channel receptors, and cargo receptors [21]. In addition, transactivation of receptor serine/threonine kinases such as ALK5 by PAR2 [14] and PAR1 [24] has been described. Both receptors display close functional interactions. For instance, PAR2 is required for PAR1 signaling [25] and both proteins show heterodimer formation [21,26]. Although both receptors are activated by different ligands (PAR1: thrombin, PAR2: trypsin/FXa), PAR2 may also be activated by thrombin under certain conditions [23]. Both receptors share a similar pattern of signaling pathways as well as overlapping spectra of functional activities under physiological and pathophysiological conditions. This is exemplified here by their involvement in inflammatory/fibrotic disorders (see Section 2.1). Finally, PAR2 and PAR1 have been found to crosstalk with the TGF-β system through various mechanisms while equivalent data for PAR3 and PAR4 are currently not available.

### 2.3. Sphingosine-1-Phosphate (S1P) as a Mediator between TGF-β and PAR Signaling

TGF-β [27,28,29], FXa via PAR2 [30], and thrombin via PAR1 [31] can induce sphingosine kinase-1 (SphK1). Sphingosine-1-phosphate (S1P) is a zwitterionic signaling lipid which is generated via SphK1 and -2 from sphingosine derived from the ceramide metabolism [32,33,34]. S1P acts both as intracellular secondary messenger and as an extracellular ligand of five cognate G-protein-coupled S1P receptors, S1P1–S1P5. Upon receptor activation, S1P activates a multitude of signaling pathways and cellular processes including cell proliferation, motility and survival [32,35]. In monocytes, S1P has recently been shown to regulate expression of PAR1 and PAR4, leading to an enhanced cell activity and amplified COX-2 expression [35]. The main sources of circulating S1P in blood are endothelial cells (ECs), red blood cells and platelets [32,34,36]. Serum S1P levels have been determined in the low micromolar range and appear to persist at least in healthy human subjects independently of age and gender [34]. During conditions of elevated platelet activation such as an acute coronary syndrome, S1P can be released from human platelets in large quantities—a process which depends on thromboxane formation and the ATP-binding cassette transporter ABCC4 (MRP4) [37,38,39]. Altering S1P levels in serum or in specific tissues appears as a novel therapeutic strategy to modulate cellular functions under certain pathophysiological conditions, i.e., we could recently show that stimulating osteoblast activity via inhibition of S1P degradation by targeting the S1P lyase is a new concept to treat osteoporosis [40]. Whether modulating the S1P signaling system in lung fibrosis might attenuate this tissue damaging process is to date unclear and might provide an attractive therapeutic perspective.

## 3. Inflammatory/Fibrotic Disorders

### 3.1. Lung Fibrosis

TGF-β [41,42], PAR2 [25,43,44,45], and PAR1 [46,47,48] have all independently been implicated in the pathogenesis of lung fibrosis. Expression of PAR2 was strongly elevated in idiopathic pulmonary fibrosis (IPF) lungs and was attributable to alveolar type II cells and fibroblasts/myofibroblasts and TGF-β1 considerably enhanced PAR2 expression in human lung fibroblasts. This was pathologically relevant since FVIIa stimulated proliferation of human lung fibroblasts and extracellular matrix (ECM) production in a PAR2-dependent manner [49].

Expression and activity of matriptase, a serine protease that induces non-canonical (biased) activation of PAR2, were upregulated in IPF and bleomycin-induced pulmonary fibrosis (BPF). In cultured human pulmonary fibroblasts, matriptase expression was significantly induced by TGF-β. Furthermore, matriptase elicited signaling via PAR2, and promoted fibroblast activation, proliferation, and migration. In the experimental bleomycin model, matriptase depletion by the pharmacological inhibitor CM or by genetic downregulation diminished lung injury, collagen production, and TGF-β expression and signaling [44]. The latter observation suggests that matriptase presumably via PAR2 also controls TGF-β expression and signaling and that both form a positive feedback loop. The expressions of PAR2 and TGF-β1/SMAD-mediated inflammation and apoptosis in BPF could be reduced by the drug, daidzein, which exhibits antifibrotic effects [50].

In IPF, TGF-β stimulates macrophages to induce fibroblast migration, differentiation and secretion of collagen and these profibrotic effects were partially mediated by PAR1. In turn, fibroblast PAR1 contributes to TGF-β activation and production. The macrophage-dependent induction of PAR1-driven TGF-β activation was mediated by FXa [51]. Similarly, the absence of PAR1 signaling affords protection from BPF [52] and was accompanied by significant reductions in pulmonary levels of the potent PAR1-inducible profibrotic and proinflammatory mediators such as TGF-β1, connective tissue growth factor (CTGF), and monocyte chemoattractant protein-1 (MCP-1) [52]. The observations that: (i) inhibition of SphK1 attenuates S1P generation and TGF-β secretion, as well as Smad2 phosphorylation in lung tissue in a BPF mouse model; (ii) bleomycin-induced expression of SphK1 in lung fibroblasts in vitro is TGF-β dependent [53]; and (iii) SphK1 is essential for thrombin-induced PAR1 signaling [31], together suggest that PAR1 facilitates TGF-β’s profibrotic effects through an increase in SphK activity and elevation of intracellular S1P.

In acute lung injury (generated by ventilator-induced lung edema), thrombin and other agonists of PAR1 activate TGF-β in an α(v)β6 integrin-specific manner. This effect is PAR1-specific and is mediated by RhoA and Rho kinase [54].

### 3.2. Kidney Fibrosis

In focal segmental glomerulosclerosis, inhibition of PAR2 signaling by systemic administration of FSLLRY-NH_2_ attenuated amplification of proinflammatory cytokines and exaggerated TGF-β1, thereby improving worsened renal functions and glomerular injury [55]. In the progressive renal disease IgA nephropathy, PAR2 activation induced a significant upregulation of TGF-β gene and protein expression in both mesangial and tubular cells. The authors suggest that PAR2 expressed by renal resident cells and activated by either mast cell tryptase or FXa may induce extracellular matrix deposition modifying the plasminogen activator inhibitor-1 (PAI-1)/tissue plasminogen activator (t-PA) balance and inducing TGF-β expression [56].

PAR2 synergizes with the TGF-β signaling pathway to contribute to renal injury and fibrosis [14]. A well-established pathway implicated in the progression of fibrosis is the induction of CTGF by TGF-β, which involves regulation by SphK1 and results in the accumulation of ECM proteins in the glomerulus [28]. Likewise, SphK2 plays an important role in kidney fibrogenesis by modulating TGF-β signaling [57]. Interestingly, CTGF can also be induced by PAR2-activating peptide (PAR2-AP) and CTGF was synergistically increased when PAR2-AP was combined with TGF-β stimulation. Consistent with these findings, treating human proximal tubular epithelial cells with PAR2-AP induced SMAD2 phosphorylation. The SMAD2 phosphorylation and CTGF induction by PAR2-AP required signaling via a SB431542-inhibitable component (presumably ALK5) and EGFR, suggesting that PAR2 utilizes transactivation mechanisms to initiate fibrogenic signaling [14]. Human kidney HK-2 cells express PAR1 and thrombin activation of this receptor has been reported to upregulate the TGF-β-mediated expression of ECM proteins, suggesting a possible pathogenic role for PAR1 signaling by thrombin in acute renal injury [58].

### 3.3. Liver Fibrosis

TGF-β [59] and PAR2 [60] have independently been associated with pathogenesis of liver fibrosis. TGF-β is a strong activator of hepatic stellate cells (HSCs) and HSC themselves produce TGF-β upon activation [61,62]. In HSCs, TGF-β1 induced expression of Col α1(I) and Col α1(III) via SphK1, which was mediated by intracellular S1P, exerting its effects in a S1P receptor-independent manner [63]. Notably, PAR2 stimulates TGF-β1 protein production and secretion by human HSCs [64]. Using shRNA-mediated knockdown of PAR2 in the HSC line LX2, our group showed that PAR2 activation via trypsin and PAR2-AP promotes secretion of the metalloproteinase ADAMTS1 and various MMPs [65]. Since ADAMTS1 is involved in TGF-β bioactivation in the liver, it is conceivable that PAR2 controls TGF-β signaling not only at the level of transcription but also by facilitating activation/processing of its precursor, latency-associated peptide-TGF-β (LAP-TGF-β) to the mature TGF-β protein.

### 3.4. Cardiac Fibrosis

In cardiac fibroblasts, cell migration and proliferation were increased upon FXa stimulation along with upregulation of TGF-β1 and H_2_O_2_ production. These data suggest that FXa plays an important role in the fibrotic process that could lead to cardiac fibrosis, and that profibrotic signaling is accelerated by FXa and PAR2 [66]. Sonin and colleagues studied the contribution of PAR1 activation on cardiac fibrosis and left ventricular (LV) remodeling in a rat model of myocardial ischemia–reperfusion injury. They found in isolated cardiac fibroblasts, which are responsible for promoting adverse LV remodeling and the development of ischemic cardiomyopathy, and in three-dimensional (3D) cardiac tissue models that PAR1 inhibition attenuated LV dilation and improved LV systolic function of the reperfused myocardium at 28 days. PAR1 inhibition also abolished thrombin-mediated ERK1/2 phosphorylation, TGF-β and type I procollagen production, MMP2/9 activation, myofibroblast transformation in vitro, and abrogated the remodeling of 3D tissues induced by chronic thrombin treatment [67]. Interestingly, S1P and SphK are also critical for TGF-β-stimulated collagen production by cardiac fibroblasts [29]. This process involves “inside-out” S1P signaling, whereby S1P produced intracellularly by SphK1 is released and acts in an autocrine/paracrine fashion to activate S1P2 receptor and increase collagen production [29].

### 3.5. Wound Healing

PAR2 has been found to stimulate TGF-β1 protein production and secretion by HaCaT keratinocytes [68]. The PAR2-dependent cellular effects of FXa led to fibroblast proliferation, migration, and differentiation into myofibroblasts, followed by the expression of TGF-β and fibronectin as well as the secretion of MCP-1 and IL-6. FXa facilitated wound healing (as assessed by wound scratch assay) in a PAR2- and ERK1/2-dependent manner. These results support the notion that, beyond its role in coagulation, FXa-dependent PAR2 cleavage might play a role in the progression of tissue fibrosis and remodeling [69]. Cultured human fibroblasts from scars exposed to TGF-β1 expressed a myofibroblast phenotype associated with overexpression of PAR2, while PAR1 expression was unaffected [70]. Similar to PAR2, PAR1 activation in vitro stimulated TGF-β production in keratinocytes and led to increased proliferation and ECM production, but not migration, of human dermal fibroblasts [71]. Moreover, thrombin-induced PAR1 transactivation of EGFR in keratinocytes and the downstream phosphorylation of the SMAD2 linker (SMAD2L) region. This thrombin-induced phosphorylation was mediated by ERK1/2, occurred at Ser250 but not Ser245 and Ser255 of SMAD2, and resulted in increased PAI-1 mRNA expression and keratinocyte migration. Thrombin-mediated PAI-1 expression and migration via EGFR transactivation may be a key pathway in skin wound healing [16].

## 4. Cancer

TGF-β [5,6,7,8,10,72], PAR2 [67,73,74,75,76,77,78,79,80,81,82,83,84,85,86,87,88,89] and PAR1 [78,86,87,90,91] have been implicated in driving proliferation, EMT, cell migration, invasion, and metastasis of cancer cells. However, a potential functional interaction between TGF-β and PAR signaling in controlling these processes has not been anticipated until our own analysis. We previously demonstrated the essential role of ERK for TGF-β- and PAR2-mediated cell motility in pancreatic tumor cells [92]. Along the same lines, analysis of signal transduction pathways activated upon PAR2 stimulation in HaCaT keratinocytes showed an involvement of ERK1/2 and profound EGFR transactivation, leading to secretion of TGF-β1 [68]. Interestingly, SphKs and S1P are critical for TGF-β-induced ERK1 and ERK2 activation and promotion of migration and invasion of esophageal cancer cells [27]. Moreover, TGF-β-mediated induction of SphK1 may have a role in human breast cancer cell bone metastasis [93]. Both TGF-β and PAR2 stimulate IL-8 release in pancreatic cancer cells and the IL-8 has been shown to affect these cells in an autocrine manner and cancer-associated fibroblasts in a paracrine manner [94]. Thus, the IL-8 signal might contribute to tumor progression and characteristic fibrosis in pancreatic cancer by inducing neo-angiogenesis through activation of the vascular endothelial growth (VEGF) factor pathway and enhancement of the activity of MMP2 and MMP9, which in turn increases the metastatic activity of the underlying malignancy [95].

TGF-β has a regulatory role in PAR1 expression, and PAR1 expression promotes tumor growth, angiogenesis and osteoclast differentiation in giant cell tumor of bone [96]. TGF-β also induces PAR1 expression in A549 lung adenocarcinoma cells, which in turn increases the sensitivity of these cells to thrombin signaling, which is consistent with the finding that TGF-β pre-stimulation promotes increased migratory potential of A549 cells to thrombin. TGF-β-mediated PAR1 upregulation is accompanied by increased expression of integrin αv and β6 subunits. These data have important implications for our understanding of the interplay between coagulation and TGF-β signaling responses in lung cancer [97].

In turn, platelet activation with a PAR1 agonist triggers TGF-β secretion, which induces EMT of SW620 human colon cancer cells via the downregulation of miR-200b expression [98]. Moreover, activation of PAR1 and PAR2 with PAR1-AP and PAR2-AP, respectively, led to activation of adventitial fibroblasts from rat aorta, including their proliferation and differentiation, ECM synthesis, as well as production of TGF-β, IL-6 and MCP-1 [99]. Platelet activation by PAR1, PAR4, and collagen receptors increased TGF-β1, VEGF, and TSP1 secretion in patients with breast cancer [100].

## 5. Types of TGF-β–PAR2 and TGF-β–PAR1 Interactions 

The TGF-β/ALK5 and PAR2 or PAR1 ligand–receptor systems interact at various levels and mechanisms including mutual regulation of ligand–ligand, ligand–receptor, and receptor–receptor at both the transcriptional/post-transcriptional and receptor transactivation levels. The term “receptor transactivation” has been redefined as “the agonist occupancy of its cognate GPCR complex which leads in a relatively short time and in the absence of de novo protein synthesis to the activation of and cytosolic generation of the immediate downstream product(s) of a second cell surface protein kinase receptor” and therefore excludes the gene to mRNA to protein sequence [101].

### 5.1. Regulation of TGF-β, TGF-β Receptors, and SMAD Proteins by PAR1 and PAR2 and their Ligands

FXa via PAR2-dependent cellular effects led to TGF-β expression, fibroblast proliferation, migration, and differentiation into myofibroblasts [69]. PAR2 stimulation triggered secretion of TGF-β1 in HaCaT keratinocytes involving EGFR transactivation and ERK1/2 activation [68], and TGF-β1 synthesis and secretion in the liver [64]. Trypsin- or PAR2-AP-activated PAR2 expressed on LX2 cells induced secretion of ADAMTS1 and some MMPs [65]. As ADAMTS1 controls activation of TGF-β in liver tissue [102], it is conceivable that serine proteases/PAR2 also control signaling by TGF-β through facilitating its conversion from the LAP-TGF-β precursor to the mature protein. 

Thrombin (via PAR1) stimulated TGF-β protein in wild-type mouse osteoblasts, but not in the corresponding PAR1 null osteoblasts [103]. The expression of PAR1 is regulated by thrombin that induced the expression of TGF-β1 to promote airway remodeling via PAR1 in ovalbumin-allergic rats [104] while treatment with a thrombin-inhibitor or a PAR1 antagonist had the opposite effect. Thrombin and other agonists of PAR1 activate TGF-β in an αvβ6 integrin-specific manner. This effect is PAR1-specific and is mediated by RhoA and Rho kinase [54]. Moreover, PLGn/plasmin, probably plasmin, through PAR1 and subsequent signaling cascade including PI3K and Akt can facilitate the production/secretion of TGF-β3 in astrocytes [105]. Finally, PAR2 activation and TGF-β signaling cooperate to downregulate microRNA-34a (miR-34a). MiR-34a is negatively regulated by PAR2 and mediates the autocrine signaling by PAR2 in colon cancer cells. Interestingly, PAR2 activation-induced downregulation of miR-34a is mediated in HT-29 colonic cancer cells by TGF-β [106].

Thrombin stimulated elongation of glycosaminoglycan chains and increased proteoglycan core protein expression in vascular smooth muscle cells and these responses were blocked by the ALK5 antagonist, SB431542 and ALK5 siRNA knockdown, as well as PAR1 antagonists. Thrombin stimulated increased C-terminal phosphorylation of SMAD2 (phospho-SMAD2C), and the response was blocked by SB431542 and JNJ5177094. A proteolytically inactive thrombin mimetic, thrombin-receptor-AP, also stimulated an increase in cytosolic phospho-SMAD2C [24]. Receptor transactivation of a SB431542-inhibitable receptor by PAR2 has also been previously suggested for PAR2-AP-mediated induction of CTGF which involves SMAD2 activation [14]. This shows that C-terminal SMAD phosphorylation can occur as a result of PAR1 activity and subsequent transactivation of ALK5. In contrast, in ECs, acute and long-term pretreatment of ECs with thrombin or PAR1 peptide agonist *suppressed* the TGF-β-induced serine phosphorylation of SMAD2. Furthermore, activation of PAR1 led to a profound and spread cytosolic clustering formation of SMAD2/3 and markedly prevented SMAD2/3 nuclear translocation evoked by TGF-β1 [107]. Moreover, thrombin via PAR1 induced the internalization of endoglin (a TGF-β coreceptor) and TβRII but not type I receptors in human ECs, an effect that was mediated by protein kinase C-ζ. Likewise, PAR1 in its inactive unligated state functions as a scaffold for TβRII to downregulate TGF-β signaling, and thereby promote embryonic stem cell (ESC) transition to functional ECs. The PAR1 scaffold function in ESCs has been suggested to be an essential mechanism for dampening TGF-β signaling and regulating ESC differentiation [108].

We have previously shown in pancreatic cells and HaCaT keratinocytes that PAR2 also promotes ALK5 expression and TGF-β1-induced SMAD3C phosphorylation, SMAD-mediated transcriptional activation and gene expression [109]. Whether PAR2 controls ALK5 expression at the level of de novo synthesis or at the level of protein stability, which represents a prominent mode of regulating ALK5 activity [110], needs to be further explored. It may also be possible that PAR2 functions as a chaperone that mediates ALK5 glycosylation and anterograde transport to the cell surface. Such a chaperone function of PAR2 has previously been described for anterograde transport of PAR4 [111]. In favor of this scenario are our recent observations that G protein-calcium signaling is dispensable for the TGF-β-promoting effect of PAR2 and, even more importantly, that PAR2 and ALK5 can physically interact with each other [92].

As mentioned above, PAR2 can transactivate ALK5 in renal cells. However, at least in PDAC-derived cells, we could not find any indication for ALK5 transactivation by PAR2 since both trypsin and PAR2-AP treatment of these cells failed to induce SMAD2/3C phosphorylation [112].

GPCRs can transduce signals not only through C-terminal but also through linker region phosphorylation of SMADs. Linker region phosphorylation arises due to activation of kinases including those downstream of the transactivation of the EGFR [14,15,16].

### 5.2. Regulation of PAR1 and PAR2 Expression and Activation by TGF-β

TGF-β regulates both PAR2 and PAR1 at the transcriptional level. The increase in gene expression of PAR2 is dependent on activation of p38 MAPK, ERK1/2 MAPK and phosphatidyl-inositol-3 kinase (PI3K), but is SMAD4-independent manner [113]. Results from our laboratory have shown that both receptors are controlled in a p38 MAPK and ERK1/2 MAPK, and Src-dependent manner, but are differentially affected by Rac1b, a splice variant of the small GTPase, Rac1 [114]. TGF-β is a positive regulator of PAR1 expression in A549 cells, and PAR1 rendered these cells more sensitive to thrombin signaling. Finally, TGF-β stimulation promoted the migratory activity of A549 cells to thrombin stimulation [97].

TGF-β promotes both its own synthesis [115] and that of serine proteinases, e.g. matriptase that subsequently can activate PAR2 via biased signaling [44]. Moreover, TGF-β1 treatment of PDAC cells resulted in the production of proteinases (of as yet unknown identity) that release from dually red/green-tagged PAR2 the N-terminal red fluorescent protein (RFP) tag, which was then visualized as a “green” receptor that remained predominantly at the cell surface. In contrast, trypsin treatment not only released the N-terminal RFP tag from PAR2, turning it “green”, but also triggered receptor internalization. These data indicate that in PDAC cells TGF-β1 is able to induce enzymes that cause autocrine cleavage/activation of PAR2 without driving PAR2 internalization [112].

In endometrial stromal cells TGF-β induced PAR2 protein and TGF-β and PAR2 synergize in secretion of IL-6 with TGF-β1 pretreatment dose-dependently enhancing the PAR2-AP-induced increase in IL-6 secretion. Treatment of endometrial stromal cells with SB431542 inhibited both TGF-β1 stimulation of PAR2 gene expression and PAR2-AP-induced IL-6 secretion, suggesting that both effects are mediated by ALK5 [113].

## 6. Conclusions and Potential Therapeutical Implications

In this review, we have assembled the various components (ligand, receptor, and intracellular signal transducer) of the TGF-β and PAR signaling pathways and the types of interactions between them. Not surprisingly, the majority of these interactions were described in physiological and pathophysiological conditions known to involve TGF-β and excessive matrix production such as wound healing, fibrosis, and cancer. The majority of interactions occur between ligands and receptors but in the case of TGF-β may also directly target the SMAD intracellular signal mediators through intermediate EGFR transactivation [14,15,16]. With respect to the mechanism, receptor transactivation, transcriptional/post-transcriptional regulation of gene expression, protein processing via induction of proteinases, and most likely regulation of protein stability and protein–protein interactions/chaperone activity are involved. These mutual interactions between PAR2/PAR1 and TGF-β signaling may eventually result in a vicious cycle (circulus vitiosus) that exacerbates profibrotic and protumorigenic effects [116]. To disrupt such a TGF-β autostimulatory loop at several levels (receptor activation/signaling, LAP-TGF-β synthesis, and bioactivation of LAP-TGF-β) and thus block unwanted TGF-β-dependent responses—but retain the beneficial ones—it might be sufficient to inhibit either PAR2 or PAR1 in addition, or alternatively, to blocking TGF-β signaling components directly [9,117]. For PAR1, vorapaxar and drotrecogin-alfa are approved PAR1-targeted therapeutics, but safety concerns have limited the clinical use of vorapaxar and questions regarding the efficacy of drotrecogin-alfa led to its withdrawal from the market [118]. Another PAR1 antagonist that is in development is atopaxar [119] which has shown promising results in Phase II trials in patients with acute coronary syndrome and high-risk coronary artery disease. The development of small-molecule non-peptide PAR2 antagonists has proven challenging. Another compound, K-14585, inhibits PAR2-dependent calcium and pro-inflammatory signaling but does not attenuate MAPK signaling [120]. GB88 is another recently developed potent PAR2 antagonist that blocks PAR2 activation by endogenous proteinase agonists as well as a PAR2-AP and can effectively attenuate inflammation in a rat model of colitis [121]. Interestingly, similar to K-14585, GB88 is a signal pathway-specific antagonist that inhibits PAR2-induced intracellular calcium release, cyclic AMP stimulation, receptor internalization and pro-inflammatory cytokine release without affecting PAR2-mediated MAPK phosphorylation [122,123]. Other PAR2 antagonists are likely to share this property of “biased” antagonism [124].
